# Editorial: Sex Hormones and Gender Differences in Immune Responses

**DOI:** 10.3389/fimmu.2019.01076

**Published:** 2019-05-09

**Authors:** Elena Ortona, Marina Pierdominici, Virginia Rider

**Affiliations:** ^1^Center for Gender Specific Medicine, Istituto Superiore di Sanità, Rome, Italy; ^2^Department of Biology, Pittsburg State University, Pittsburg, KS, United States

**Keywords:** immune response, sex hormones, autoimmunity, infections, allergy

In general, females have stronger innate and adaptive (humoral and cellular) immune responses in comparison to males. The factors responsible for the stronger immune response in females than males may be due to biologic factors (i.e., sex differences, such as genetic and epigenetic factors, sex hormones) and to psychosocial factors (i.e., gender differences). Our aim in assembling this Research Topic was to highlight the current understanding of the role played by sex hormones (i.e., androgens, progesterone, prolactin, and estrogens) and their receptors in modulating the immune response. In addition, we wanted to highlight the possibility that sex differences could alter the susceptibility and/or the severity of autoimmune and infectious diseases. A better comprehension of sex hormone-immune response interactions could lead to innovative and readily available therapeutic interventions, such as hormone antagonists or agonists, as new approaches to manage immune-mediated diseases.

The collection is comprised of a series of reviews and original research papers underlining the role of sex hormones in immune response modulation as well as hormone influence on autoimmune diseases, infections and allergy. Borba et al., discuss the role of prolactin in immune system modulation and the involvement of prolactin in the pathogenesis and activity of several autoimmune disorders. The Authors describe the evidence for dopamine as an effective inhibitor of prolactin secretion and suggest that dopamine agonists could represent a promising novel therapy for autoimmune patients. Moulton summarizes a large body of evidence for estrogenic effects in the adaptive immune response in health and autoimmunity with an emphasis on systemic lupus erythematosus (SLE). Gubbels Bupp and Jorgensen provide a comprehensive review on the action of androgens, working through their receptors to dampen or alter immune responses. Androgens affect the onset of autoimmune diseases as well as disease progression. Gubbels Bupp et al. provide a timely and interesting review describing the age- and sex hormone-related changes to innate and adaptive immunity. Their review highlights the importance of age-and sex-associated changes in the immune system and the subsequent impact on the onset of autoimmunity, cancers, and the efficacy of vaccination and cancer immunotherapy. In an opinion article by Taneja, interactions among environmental factors (diet, infections, cigarette smoke) and sex hormones are postulated to influence immune responses. Bereshchenko et al. point to the importance of possible interactions between glucocorticoid and sex steroid receptors that could underpin the sexual disparity of autoimmune diseases. Additional research is necessary to investigate possible “cross talk” among steroid receptors to identify interacting signaling pathways that may be crucial in fully understanding the onset of autoimmune diseases and gender differences. Two reviews in the collection focus on sex hormones and viral infections. Kadel and Kovats discuss evidence that sex differences exist in both respiratory homeostasis and viral infections owing to differential regulation by sex hormones in innate immune cells in the lungs. Additional complications of sex differences in the respiratory system occur because of disparate influence of sex hormones on the proinflammatory/effector phase and/or the resolution/tissue repair phase in innate cells. These differences ultimately contribute to the host's ability to respond to respiratory viral infections. Ruggieri et al. discuss the effects of sex hormones on the immune system response to Hepatitis B and C virus infections. Included in their review is evidence for direct sex hormone influence on viral activity. Shah and Newcomb discuss data supporting differences in allergic responses between males and females. Fluctuations in sex hormones during puberty, menstruation, pregnancy, and menopause, may alter the symptoms and severity of asthma.

The second part of the collection includes original research articles that explore novel aspects of sex hormone action in immune responses.

Two research articles focus on estrogen receptors in immune modulation and their impact on autoimmune diseases. Rider et al. investigated cell signaling changes in human SLE T cells treated with estradiol and the estrogen receptor α antagonist, Fulvestrant, comparing the effects of blockading the action of estrogen receptor α in order to identify signaling pathways that could contribute to improved disease activity in women with SLE. The Authors identified alterations in several pathways including T helper cell differentiation, steroid receptor signaling, ubiquitination, and sumoylation. In their research article, Dupuis et al. provide new insight regarding the anti-inflammatory effects of the phytoestrogen silibinin. Silibin binds to estrogen receptor β in T lymphocytes from both female and male healthy subjects and patients with rheumatoid arthritis. Silibinin induces apoptosis, inhibits proliferation, and reduces expression of the pro-inflammatory cytokines IL-17 and TNF-α, suggesting a potential role for this phytoestrogen in rheumatoid arthritis management.

Adverse pregnancy outcome related to autoimmunity represents a hot topic in translational research. In this regard, Fredi et al. evaluated the risk factors for adverse pregnancy outcomes in patients with antiphospholipid antibodies positivity. The Authors observed maternal and fetal complications in some antiphospholipid antibodies—positive patients and a higher risk of adverse pregnancy outcome in patients with a previous thrombosis. Research on a similar topic by Truglia et al. focused at utilizing new and sensitive approaches to identify antiphospholipid antibodies in patients with obstetrical antiphospholipid syndrome who are negative when tested using conventional laboratory markers.

Sex differences in infection was the topic of two original research articles. Scalerandi et al., using a bacterial model of prostate inflammation, showed an intriguing effect of testosterone in promoting inefficient, anti-inflammatory neutrophils that prolonged bacterial inflammation, generating a pathogenic environment for several conditions. Celestino et al. observed that female mice are less susceptible than males to mouse-adapted influenza virus (A/PR8/H1N1). Their analysis of the underlying mechanism that contributes to the sex disparities suggested that the female mice generate higher total antioxidant power in their sera and lungs when compared with male mice.

Understanding how sex influences immunity is still in its infancy. However, recent evidence (1), including the papers of this collection, indicate that components of both innate and adaptive immunity are differently regulated in females and males. Sex differences contribute to differences in susceptibility and severity of immune-mediated and infectious diseases, and malignancies (1, 2). Sex hormones can affect different steps in immune processes. Thus, the complexity of endocrine-immune interaction represents a recurrent theme in the papers comprising this collection. Age-specific responses may also influence immune-hormone interactions. An integrated approach focused at analyzing the relationships among sex hormones, sex chromosomes and immune related genes is needed to better understand gender differences in immune response (3, 4).

We hope that this collection of primary research papers and review articles will prove useful to investigators interested in the current state-of-the-art research into sex hormones and immune responses.

## Author Contributions

All authors listed have made a substantial, direct and intellectual contribution to the work, and approved it for publication.

### Conflict of Interest Statement

The authors declare that the research was conducted in the absence of any commercial or financial relationships that could be construed as a potential conflict of interest.
